# Influence of condensation temperature on selected exhaled breath parameters

**DOI:** 10.1186/1471-2466-5-10

**Published:** 2005-09-01

**Authors:** Matteo Goldoni, Andrea Caglieri, Roberta Andreoli, Diana Poli, Paola Manini, Maria Vittoria Vettori, Massimo Corradi, Antonio Mutti

**Affiliations:** 1 National Institute of Occupational Safety and Prevention, Research Center at the University of Parma, Parma, Italy; 2 Department of Clinical Medicine, Nephrology and Health Sciences, University of Parma, Parma, Italy

## Abstract

**Background:**

The effects of changes in cooling temperature on biomarker levels in exhaled breath condensate have been little investigated. The aim of the study was to test the effect of condensation temperature on the parameters of exhaled breath condensate and the levels of selected biomarkers.

**Methods:**

Exhaled breath condensate was collected from 24 healthy subjects at temperatures of -10, -5, 0 and +5 C degrees. Selected parameters (condensed volume and conductivity) and biomarkers (hydrogen peroxide, malondialdehyde) were measured.

**Results:**

There was a progressive increase in hydrogen peroxide and malondialdehyde concentrations, and condensate conductivity as the cooling temperature increased; total condensate volume increased as the cooling temperature decreased.

**Conclusion:**

The cooling temperature of exhaled breath condensate collection influenced selected biomarkers and potential normalizing factors (particularly conductivity) in different ways ex vivo. The temperature of exhaled breath condensate collection should be controlled and reported.

## Background

Exhaled breath condensate (EBC) is a biological fluid that mainly consists of water, but also contains small droplets of airway lining fluid. Much of the interest of EBC lies in the fact that its collection is totally non-invasive and does not lead to any discomfort or risk [1]. It has been used to assess inflammatory airway diseases such as asthma [2], chronic obstructive pulmonary disease [3], lung cancer [4], interstitial lung disease [5] and acute respiratory distress syndrome [6], and has recently also been extended to the biological monitoring of workers exposed to cobalt and tungsten [7].

EBC contains both volatile and non-volatile substances. Volatile or semi-volatile substances have appreciable vapour pressure at body temperature, and can therefore be breathed out as gases; furthermore, volatile substances in gaseous phase can be dissolved in condensed water during EBC collection depending on their physico-chemical properties [8]. Non-volatile substances, such as salts and proteins, are mainly expired in small droplets, and further diluted with exhaled water vapours [8,9]. It is thought that the droplets are formed as a result of random convective processes, and may not be directly related to water vapour production.

This has raised the question of variable droplet dilution, and given rise to some concerns regarding the interpretation of EBC biomarkers on the basis of their absolute concentration [9]. Some authors have suggested normalising for ion concentrations (Na^+^, Cl^-^, K^+^) or urea, assuming that they are equally concentrated in the airway lining fluid and serum of healthy and diseased subjects [8,10], and conductivity measurements of lyophilised EBC have also been proposed as a normalization factor [11]. On the other hand, the use of non-volatile parameters to normalise the volatile or semi-volatile compounds in EBC could ignore their condensation pathways as their *ex vivo *evaporation from the airways is different from droplet condensation in EBC collection systems. Ideally, a normalisation factor should be identified among substances with the same physical and chemical characteristics (i.e., volatility and solubility) as the measured parameters.

Volatility is generally assessed by means of a status diagram, which represents the relationship between the compounds' vapour pressure and temperature [12]. In a condensing system, the vapour pressure of volatile compounds depends on condensation temperature, and so the physical and chemical properties of exhaled compounds can be weighted by changing the condensation temperature. This may be particularly relevant in the case of EBC, in which the condensation of each substance may be affected by the presence of other compounds in the same solution.

A new type of condenser has been specifically designed to control the temperature of EBC collection, and test the effect of different condensation temperatures on the recovery of selected biomarkers (hydrogen peroxide, malondialdehyde). We also measured the conductivity after EBC lyophilisation, a parameter that reflects the overall concentration of salts. A rigorously precise study design controlling these aspects may represent a major advance in our understanding of condensation mechanisms, and in the validation of EBC as a suitable source of biomarkers reflecting the pathobiology underlying lung diseases.

## Methods

### Subjects

EBC was collected from 24 healthy non-smokers (table 1): i.e. asymptomatic and non-atopic individuals with normal spirometry results who showed no bronchial hyper-responsiveness to methacholine. The study was conducted in conformity with the declaration of Helsinki and was approved by the Ethical Committee of the University of Parma. All of the subjects gave their informed written consent.

**Table 1:** Characteristics of the study subjects. Data are expressed as mean ± SD.

**Number of Subjects**	24 (13 M; 11 F)
**Age, Years**	30 ± 4
**Smokers/Ex-Smokers/Non Smokers**	0/3/21
**FVC, % of predicted**	108.4 ± 10.4
**FEV_1_, % of predicted**	103.7 ± 11.4
**FEV_1_/FVC, % of predicted**	81.9 ± 6.3

### Study design

The number of volunteers was exactly the same as the total number of possible sequence combinations of 4 temperatures (4! = 24). EBC was randomly collected from each subject using a different sequence of the four temperatures. The duration of the clinical part of the study was about two weeks in order to avoid the possible influence of climatic changes on EBC parameters. In order to assess inter-session variability, 10 of the 24 subjects underwent four consecutive EBC collections at the two extremes of temperature (-10°C and +5°C).

### Collecting system

TURBO-DECCS (Transportable Unit for Research on Biomarkers Obtained from Disposable Exhaled Condensate Collection Systems) is a new and commercially available device for EBC collection (ItalChill, Parma, Italy). TURBO is a refrigerating system relying on a thermo electrical module giving rise to Peltier effect, which is activated by an electronic circuit in continuous current fed with main voltages. The cold side of the Peltier module is connected to an aluminium support shaped to house the test tube. A thermostat allows the collecting temperature to be regulated with a tolerance of ±1°C. The working temperature is adjustable from -10°C to room temperature or higher.

TURBO is supplied with DECCS, a disposable polyethylene device for collecting EBC that consists of a mouthpiece equipped with a one-way valve and saliva trap, connected to a collecting vial (50 ml) by means of a tube.

### EBC collection

The subjects were asked to breath tidally through the mouthpiece without a nose clip for 10 minutes at collecting temperatures of -10°C, -5°C, 0°C and +5°C, with a 5-min interval after every collection. The subjects collected their EBC all in the same laboratory and no one collected EBC at home. EBC from every subject was collected according to different temporal combinations of the four temperatures covering the 24 possible combinations. The subjects were strictly instructed to maintain constant tidal breathing during the test and to form a complete seal around the mouthpiece; excess saliva was periodically eliminated and the mouth was rinsed with water. Salivary contamination was excluded by means of the colorimetric detection of alpha-amylase (Infinity amylase reagent, Sigma, Milan, Italy). The EBC samples were centrifuged for 1 min at 1000 *g *immediately after collection so that all of the water droplets were driven to the bottom of the flask, and the total volumes were measured. The samples were then stored at -80°C until analysis.

### EBC analysis

#### Volume

The collected EBC volume was measured using a calibrated 200 μl pipette (Gilson International, Den Haag, The Netherlands) with an experimental error of ±10 μl.

#### Conductivity

EBC was lyophilised using a Heto FD 1.0 lyophilizer (Heto, Allerod, Denmark) and then re-suspended in ultra-pure water (Sigma, St. Louis, MO, USA). Conductivity was measured by means of an Istek 430c conductivity meter (Istek, Seoul, Republic of Korea). The Limit of Detection (LOD) of the method was about 0.1 μS/cm. Contamination of the storage vials was carefully assessed and excluded.

#### Hydrogen peroxide (H_2_O_2_)

H_2_O_2 _in EBC was measured as previously described [13] using a commercial kit (Amplex Red Hydrogen Peroxide assay kit, Molecular Probes, Eugene, USA) with a LOD of 0.01 μM. The analysis was made within 2–3 days of EBC collection after freezing at -80°C, in order to avoid the relative instability of H_2_O_2 _in EBC [14].

#### Malondialdehyde (MDA)

EBC MDA was measured by means of liquid chromatography-mass spectrometry tandem (LC-MS/MS) as previously described [15] within two weeks of EBC collection after storage at -80°C because of the relative high stability of MDA [16].

### Statistical analysis

Data distribution was assessed using the Shapiro-Wilk test. Mean values ± SD were used for the normally distributed data, and geometric means [geometric SD] for the data with a lognormal distribution. Between-group differences were calculated using one-way ANOVA for repeated measures, followed by Tuckey's *post-hoc *test using the experimental data points or their logarithms, depending on the distribution of the experimental values. Regressions were performed using the least-squares method on either the experimental data points or their logarithms, using the Pearson's correlation coefficient to test goodness-of-fit. Intra-individual variability due to repeated measures was assessed using the dummy variables method for the multiple regression analysis [17]. A significance level of 0.05 was chosen for all of the statistical tests. The data were statistically analysed using two software programmes: SPSS 12.0 (SPSS inc., Chicago, IL, USA) and PRISM 3.0 (Graphpad Sofware, San Diego, CA, USA).

## Results

Table 2 shows the inter-session variability in 10 of the 24 subjects at the two extreme temperatures (-10°C and +5°C). No statistically significant differences were found in the total volume, H_2_O_2 _level or conductivity values of the four consecutive collections.

**Table 2:** Inter-session variability of the selected parameters. t_1_, ...., t_4 _represent four consecutive EBC collections from 10 healthy subjects. No significant differences were found using repeated measures ANOVA followed by Tuckey's post-hoc test. MDA was excluded from the analysis because its reliability has been previously measured [15,16]. Mean ± SD or geometric mean [geometric SD] are also reported.

	**Total Volume (μl)**				**H_2_O_2 _(μM)**				**Conductivity (μS/cm)**			
**T**	**t_1_**	**t_2_**	**t_3_**	**t_4_**	**t_1_**	**t_2_**	**t_3_**	**t_4_**	**t_1_**	**t_2_**	**t_3_**	**t_4_**

**-10°C**	1080 ± 270	1050 ± 220	1120 ± 250	1150 ± 220	0.097 [1.85]	0.092 [1.95]	0.108 [1.97]	0.112 [1.95]	1.8 [2.26]	2.6 [2.76]	2.4 [2.75]	1.5 [2.19]
**+5°C**	610 ± 200	680 ± 230	660 ± 250	600 ± 250	0.135 [1.92]	0.118 [1.88]	0.125 [1.91]	0.140 [2.00]	6 [2.95]	4.8 [2.70]	5.5 [2.50]	6.5 [2.33]

**Figure 1:**
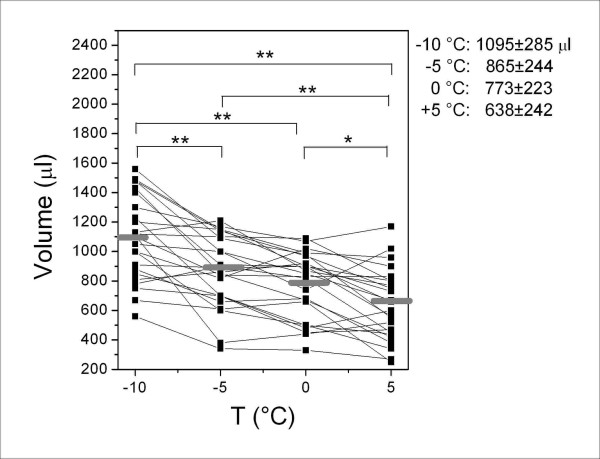
**Variation in total EBC volume collected at different cooling temperatures**. The horizontal grey lines indicate mean values. The parameters of the distributions (mean ± SD) at different temperatures and the significant differences are also reported. * = *p *< 0.05; ** = *p *< 0.01.

**Figure 2:**
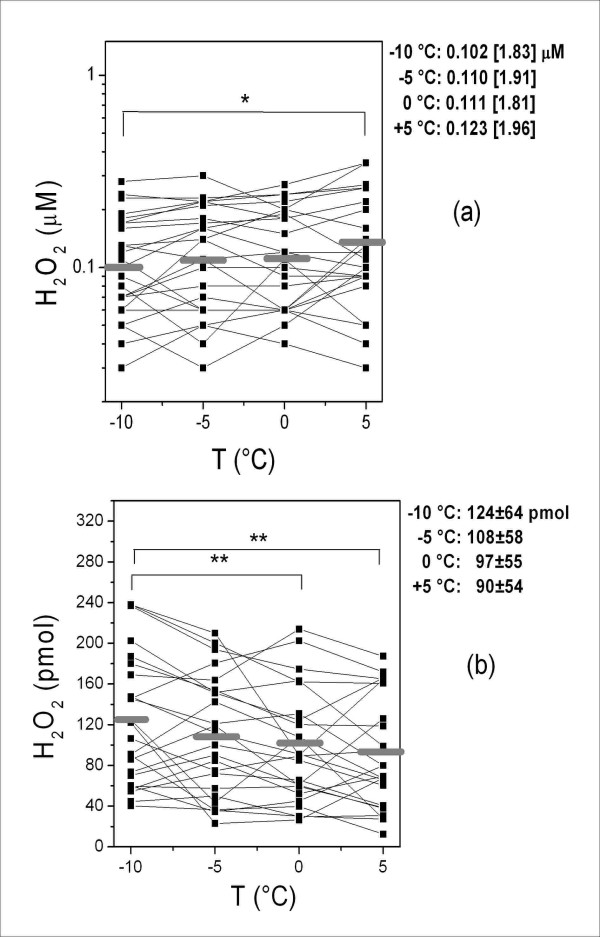
**Concentration (a) and absolute amount (b) of EBC H_2_O_2 _at different cooling temperatures**. The horizontal grey lines indicate geometric mean (a) and mean values (b). The parameters of the distributions (geometric mean [geometric SD] and mean ± SD for a and b respectively) at different temperatures and the significant differences are also reported. * = *p *< 0.05; ** = *p *< 0.01.

**Figure 3:**
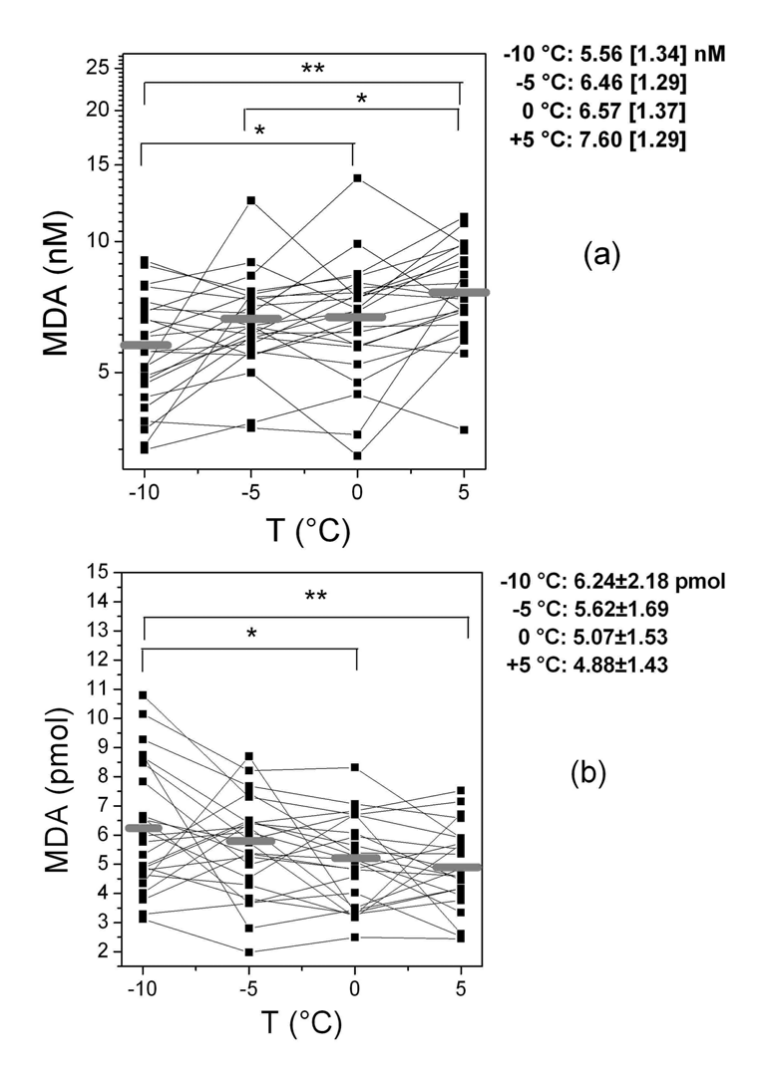
**Concentration (a) and absolute quantity (b) of EBC MDA at different cooling temperatures**. The horizontal grey lines indicate geometric mean (a) and mean values (b). The parameters of the distributions (geometric mean [geometric SD] and mean ± SD for a and b respectively) at different temperatures and the significant differences are also reported. * = *p *< 0.05; ** = *p *< 0.01.

**Figure 4:**
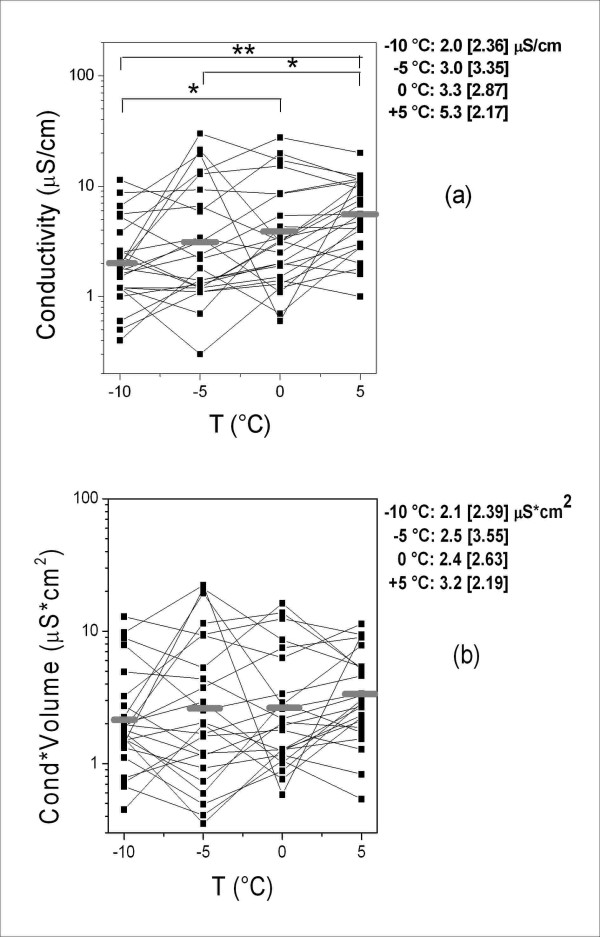
EBC conductivity (a) and EBC conductivity*EBC volume values (b) at different cooling temperatures. The horizontal grey lines indicate geometric mean values. The parameters of the distributions (geometric mean [geometric SD]) at different temperatures and the significant differences are also reported. * = *p *< 0.05; ** = *p *< 0.01.

The measurements of the variables in the samples collected at different temperatures are shown in Figures 1, 2, 3, 4, which also show their distribution parameters (mean ± SD or geometric mean [geometric SD]). There was a clear trend toward increasing EBC sample volumes with decreasing collection temperatures (Figure 1), which affected both the concentration and absolute amounts of the selected analytes to different extents.

All of the differences in volume between temperature pairs were statistically significant, except for the comparison between -5°C and 0°C. At a fixed temperature, the total volume of expired air closely correlated with the total condensed volume (r > 0.95, data not shown).

The H_2_O_2 _concentrations measured in the EBC samples collected at different cooling temperatures (Figure 2a) were significantly different between -10°C and +5°C (*p *< 0.05). The absolute amount of H_2_O_2 _(in pmol) was calculated by multiplying its concentration by the corresponding total condensed volume. As shown in Figure 2b, a progressive increase in the amount of EBC H_2_O_2 _was recorded as the cooling temperature decreased, with significant differences between -10 and +5°C and between -10 and 0°C (*p *< 0.01).

Figure 3a shows the differences in MDA concentration measured in the EBC samples collected at different cooling temperatures (between -10 and 0°C and between -5 and +5°C, *p *< 0.05; between -10 and +5°C, *p *< 0.01). Figure 3b shows that the absolute amount of MDA (in pmol) decreased with increasing temperature, with some residual statistically significant differences between -10 and 0°C (*p *< 0.05) and between -10 and +5°C, (*p *< 0.01).

There was a progressive increase in EBC conductivity values at different cooling temperatures (Figure 4a), with significant differences between -10 and 0°C, between -5 and +5°C (*p *< 0.05) and between -10 and +5°C (*p *< 0.01). These differences disappeared when conductivity was multiplied by volume (thus expressing results as μS*cm^2^), as shown Figure 4b.

On the basis of the geometric standard deviation (GSD) of the values shown in Figures 2a, 3a and 4a, conductivity was more variable (range 2.17–3.35) than either H_2_O_2 _(range 1.81–1.96) or MDA (range 1.29–1.37) concentrations.

Table 3 shows the parameters of regressions between measured variables: Pearson's correlation coefficient with its significance, and the intercept with Y axis (A) and the slope (B). The regressions were performed using the least-square method either on experimental data points or their logarithms, with Pearson's correlation coefficient being used to test the goodness-of-fit. Intra-individual variability due to repeated measures was assessed using the dummy variable method for multiple regression analysis. The results indicated a weak negative correlation between total volume and H_2_O_2 _(r = -0.29, *p *< 0.01) but, considering B values in table 3, the intra-individual contribution was not significant. There was also a negative correlation between total volume and MDA (r = -0.54, *p *< 0.01) with a similar inter-individual and intra-individual contribution. Conductivity showed a similar, albeit weaker correlation (r = -0.34, *p *< 0.01). H_2_O_2 _and MDA levels positively correlated (r = 0.53, *p *< 0.01). There was no significant correlation between H_2_O_2 _and conductivity (r = 0.1, *p *= ns), but a significant, albeit weak correlation between MDA and conductivity (r = 0.21, *p *< 0.05), with a higher intra-individual contribution to variability.

**Table 3:** Regressions between the measured variables. Regressions between the measured variables using the general model (second column) and the dummy variable method (third column). ns = not significant. Cond. = Conductivity, Vol. = Volume. r = Pearson's correlation coefficient; p = significance of r; A: intercept with Y-axis (±SD is also expressed); B: Slope of the regression line (±SD is also expressed); p (B): significance of B. The correlations refer to the concentrations of H_2_O_2 _and MDA. E_i _= dummy variable. Its value is 1 for the i-th subject, -1 for the last subject, 0 elsewhere.

**H_2_O_2 _vs Vol.**	Log (H_2_O_2_) = A + B*Volume					Log (H_2_O_2_) = A + B*Volume + Σ_i_C_i_E_i_		
	r	*P*	A	B	*p *(B)	A	B	*p *(B)
	-0.29	**<0.01**	-0.73 ± 0.08	(-2.7 ± 0.9)*10^-5^	**<0.01**	-0.88 ± 0.08	(-8.5 ± 5.2)*10^-5^	ns
**MDA vs Vol.**	Log (MDA) = A + B*Volume					Log (MDA) = A + B*Volume + Σ_i_C_i_E_i_		
	r	*P*	A	B	*p *(B)	A	B	*p *(B)
	-0.54	**<0.01**	1.01 ± 0.04	(-2.4 ± 0.4)*10^-4^	**<0.01**	1.00 ± 0.04	(-2.3 ± 0.4)*10^-4^	**<0.01**
**Cond. vs Vol.**	Log (Cond.) = A + B*Volume					Log (Cond.) = A + B*Volume + Σ_i_C_i_E_i_		
	r	*P*	A	B	*p *(B)	A	B	*p *(B)
	-0.34	**<0.01**	0.94 ± 0.13	(-5.2 ± 1.5)*10^-4^	**<0.01**	1.05 ± 0.13	(-6.5 ± 1.4)*10^-4^	**<0.01**
**MDA vs H_2_O_2_**	Log (MDA) = A + B*Log (H_2_O_2_)					Log (MDA) = A + B*Log (H_2_O_2_) + Σ_i_C_i_E_i_		
	r	*P*	A	B	*p *(B)	A	B	*p *(B)
	0.53	**<0.01**	1.05 ± 0.04	0.25 ± 0.04	**<0.01**	1.16 ± 0.09	0.37 ± 0.10	**<0.01**
**H_2_O_2 _vs Cond.**	Log (H_2_O_2_) = A + B*Log (Cond.)					Log (H_2_O_2_) = A + B*Log (Cond.) + Σ_i_C_i_E_i_		
	r	*P*	A	B	*p *(B)	A	B	*p *(B)
	0.12	Ns			Ns			ns
**MDA vs. Cond.**	Log (MDA) = A + B*Log (Cond.)					Log (MDA) = A + B*Log (Cond.) + Σ_i_C_i_E_i_		
	r	*P*	A	B	*p *(B)	A	B	*p *(B)
	0.21	**<0.05**	0.78 ± 0.02	0.06 ± 0.03	**<0.05**	0.76 ± 0.02	0.10 ± 0.03	**<0.01**

## Discussion

Although EBC is mostly water, it contains appreciable concentrations of volatile and non-volatile solutes. The presence of salts and peptides in EBC suggests a transfer of non-volatile compounds to the air phase, probably in the form of small droplets to allow the vapour stream to go through convective processes. Mathematical models based on *in vitro *experiments [18] have been developed in order to understand more about the physical phenomenon of droplet formation, and the size distribution of exhaled droplets has been characterized [19,20]. Other Authors have proposed complex mathematical models designed to account for the presence of non-volatile solutes in ambient air [21,22].

Various approaches have been proposed as a mean of normalising biomarker concentrations in EBC [8-11], including the use of endogenous non-volatile substances (ions, urea) or parameters (osmolality, conductivity after lyophilisation). One controversial assumption is that serum and airway surface lining fluid are isotonic, but this is not supported by available evidence [23-25]. Although a normalisation factor may be useful for non-volatile molecules such as proteins and electrolytes, the *ex vivo *volatility of some currently measured biomarkers in EBC is still unclear, and their normalization by any non-volatile factor could actually lead to ignore their different exhalation pathways and consequent focusing ability in EBC collecting systems.

In this study, we assessed the *ex vivo *volatility of H_2_O_2 _and MDA, which are respectively considered to be reliable biomarkers of airway inflammation and membrane peroxidation [26,27]; the volatility and solubility of H_2_O_2 _are well known in aqueous solutions [28], but little is known about MDA. We also measured total condensed volume, which reflects overall subject ventilation [29], and conductivity after EBC lyophilisation, which reflects the concentration and charge of non-volatile electrolytes [11].

As expected, total condensed volume inversely related to temperature. On a pretty constant subject expiration, the number of condensed water molecules derived from the aqueous vapour phase depends on condensation temperature; with lower temperatures condensating a larger number of water molecules. Assuming an EBC density of approximately 1 g/cm^3 ^(near to that of pure water), the increase in the number of water molecules in the selected range of cooling temperatures can be calculated using the equation:

% increase water = Volume (-10°C)/Volume (+5°C) = 1.72,

On the basis of the mean values calculated in Figure 1. This ratio is significantly different from 1 (*p *< 0.01).

The same equation (with different means and geometric means depending on the data distribution) was used to calculate the absolute amount of H_2_O_2 _and MDA. As the ratios were significantly different from 1 (1.37 for H_2_O_2 _and 1.28 for MDA, *p *< 0.01, Figures 2b and 3b), these biomarkers can be considered volatile compounds *ex vivo*. In both cases, the percentage of recovery is less than that of water. Therefore, as the condensation temperature decreased, the increase in their absolute amount was less than that of water.

The behaviour of EBC conductivity is an important comparative parameter. Like H_2_O_2 _and MDA, conductivity increased with temperature, although in this case the absolute increase was greater and inversely related to volume (Figure 4a). Assuming that EBC conductivity is proportional to mono-charged ion EBC concentration, which should represent more than 80% of the total [11], the product of conductivity by volume should be approximately proportional to the number of electrolyte molecules and, as expected, the differences between collection temperatures were no longer significant (Figure 4b). This may be due to the fact that the number of expired droplets (and therefore the absolute amount of recovered ions) is not temperature dependent, whereas the observed decrease in conductivity at lower temperatures should only depend on number of condensed water molecules with the subsequent dilution of non-volatile substances. We calculated the percent increase in salt molecules using the equation:

% increase salts molecules ≈ Conductivity*Volume (-10°C)/Conductivity*Volume (+5°C) = 0.66,

on the basis of the geometric mean values shown in figure 4b. The ratio is not significantly different from 1.

On the basis of our data, H_2_O_2 _and MDA should both be considered volatile substances *ex vivo*, whereas conductivity should not. The normalisation of these volatile substances by non-volatile compounds or parameters would therefore not take into account their specific excretion pathway. Further, the high variability of conductivity (Figure 4a) suggests that its level in EBC is not regulated by a specific biological elimination mechanism, but depends on a random process, that would make its use as a normalisation factor hazardous. Effros *et al*. [9] suggested that volatile compounds should be measured in the gas phase of expiration rather than in EBC, but our findings show that, although the determination of low-volatility compounds is influenced by the condensation temperature, their determination in EBC is not nonsensical provided that the EBC collection temperature is fixed: at the relatively high condensation temperature of +5°C, about 70% of the total molecules measured at -10°C were present in solution.

The intra-session variability study (Table 2) showed that temperature-related differences in collected marker levels can not be attributed to intra-individual variability. MDA reliability was not assessed as it has already been demonstrated [15,16].

The between-variable correlations and regressions gave further information concerning the mutual relationships of the compounds. When regression was calculated without making any distinction for repeated measures, the contribution of temperature was mixed with other possible contributions, such as that of ventilatory volume, which closely correlated with the total collected volume at a fixed temperature. Isolation of the intra-individual effect by means of the dummy variable method distinguished the effect of temperature from the other contributions as the ventilatory volume of the same subject was kept constant at the different temperatures, and made it possible to estimate its effect on the parameters describing the regression.

EBC H_2_O_2 _concentrations showed a weak negative correlation with EBC total volume (r = -0.29), but this was not significant when only the intra-individual effect was considered (Table 3). Although the temperature-induced variations in volume did not correlate with the variations in H_2_O_2 _concentration, the inter-individual variability in volume due to other contributions moderately affected H_2_O_2 _concentrations. On the contrary, EBC MDA concentration negatively correlated with total EBC volume (r = -0.54), with a similar contribution of inter-individual and intra-individual effect (Table 3). As a result, total volume could be a relevant parameter, particularly if the EBC biomarker correlates with EBC volume and the effect of condensation temperature is ruled out. When sampling occurs at a constant temperature, any inter-individual difference in EBC volume can be ascribed to total ventilation volume. Under these circumstances, the use of total EBC volume as a covariate could normalise the effect of total ventilation volume on biomarker concentrations.

H_2_O_2 _and MDA positively correlated (r = 0.53). These data suggest that, in addition to their volatility, hydrogen peroxide production and lipid peroxidation could also be related processes *in vivo *in healthy subjects, as already observed in subjects affected by respiratory tract inflammation [30].

Finally, although there was no correlation between H_2_O_2 _concentration and conductivity values, thus reinforcing the idea that non-volatile compounds and H_2_O_2 _in EBC have different physico-chemical properties, a very weak positive correlation was found between MDA and conductivity (r = 0.21): this suggests that a slight contribution to the total concentration of MDA in EBC could derive from MDA-containing droplets. In fact, the lack of correlation between conductivity and volatile components indicates that non-volatile ions reflect the number of airway lining fluid droplets joining the vapour stream, a mechanism that would complement evaporation. The latter seems to be the main determinant of H_2_O_2 _(which is volatile) and MDA (which is also slightly volatile) content in EBC.

## Conclusion

On the basis of the present study, we suggest that:

1. the temperature of EBC collection should be controlled and reported;

2. cooling temperatures should be chosen on the basis of analytical needs (required EBC volumes, sensitivity of the method, etc.);

3. water is the main variable dilution factor, and so total condensed volume should be recorded;

4. the cooling temperature related to EBC collection may differently influence biomarkers and normalizing factors, which should belong to the same class as the analytes requiring normalization (e.g., in terms of relative volatility and solubility).

## Abbreviations

EBC = Exhaled Breath Condensate; MDA = Malondialdehyde; LOD = Limit of Detection; LC-MS/MS = liquid chromatography-mass spectrometry tandem; TURBO-DECCS = Transportable Unit for Research on Biomarkers Obtained from Disposable Exhaled Condensate Collection Systems.

## Competing interests

The author(s) declare that they have no competing interests.

## Authors' contributions

MG: substantial contribution to conception and design, acquisition of data, analysis and interpretation of data, involved in drafting the article.

AC: substantial contribution to conception and design, collection of samples, revision of the draft critically for important intellectual content.

RA:, acquisition of data, revision of the draft critically for important intellectual content.

DP: substantial contribution to conception and design, revision of the draft critically for important intellectual content.

PM: substantial contribution to conception and design, revision of the draft critically for important intellectual content.

MVV: substantial contribution to conception and design, acquisition of data, revision of the draft critically for important intellectual content.

MC: substantial contribution to conception and design, analysis and interpretation of data, involved in drafting the article.

AM: substantial contribution to conception and design, statistical analysis and interpretation of data, involved in drafting the article, final approval of the version to be published.

## Pre-publication history

The pre-publication history for this paper can be accessed here:


